# Disentangling the mechanisms sustaining a stable state of submerged macrophyte dominance against free-floating competitors

**DOI:** 10.3389/fpls.2022.963579

**Published:** 2022-10-31

**Authors:** Sándor Szabó, Gergő Koleszár, Györgyi Zavanyi, Péter Tamás Nagy, Mihály Braun, Sabine Hilt

**Affiliations:** ^1^ Department of Biology, University of Nyíregyháza, Nyíregyháza, Hungary; ^2^ Department of Tisza Research, Centre for Ecological Research, Debrecen, Hungary; ^3^ Doctoral School of Biological Sciences, Hungarian University of Agriculture and Life Sciences, Gödöllő, Hungary; ^4^ Institute of Water and Environmental Management, University of Debrecen, Debrecen, Hungary; ^5^ Isotope Climatology and Environmental Research Centre (ICER), Institute for Nuclear Research, Eötvös Loránd Research Network, Debrecen, Hungary; ^6^ Department of Community and Ecosystem Ecology, Leibniz Institute of Freshwater Ecology and Inland Fisheries (IGB), Berlin, Germany

**Keywords:** alternative stable states, *Lemna*, multiple stressors, nitrogen, nutrients, pH

## Abstract

Free-floating and rootless submerged macrophytes are typical, mutually exclusive vegetation types that can alternatively dominate in stagnant and slow flowing inland water bodies. A dominance of free-floating plants has been associated with a lower number of aquatic ecosystem services and can be explained by shading of rootless submerged macrophytes. *Vice versa*, high pH and competition for several nutrients have been proposed to explain the dominance of rootless submerged macrophytes. Here, we performed co-culture experiments to disentangle the influence of limitation by different nutrients, by pH effects and by allelopathy in sustaining the dominance of rootless submerged macrophytes. Specifically, we compared the effects of nitrogen (N), phosphorus (P), iron (Fe) and manganese (Mn) deficiencies and an increased pH from 7 to 10 in reducing the growth of free-floating *Lemna gibba* by the rootless *Ceratophyllum demersum*. These macrophyte species are among the most common in highly eutrophic, temperate water bodies and known to mutually exclude each other. After co-culture experiments, additions of nutrients and pH neutralisation removed the growth inhibition of free-floating plants. Among the experimentally tested factors significantly inhibiting the growth of *L. gibba*, an increase in pH had the strongest effect, followed by depletion of P, N and Fe. Additional field monitoring data revealed that in water bodies dominated by *C. demersum*, orthophosphate concentrations were usually sufficient for optimal growth of free-floating plants. However, pH was high and dissolved inorganic N concentrations far below levels required for optimal growth. Low N concentrations and alkaline pH generated by dense *C. demersum* stands are thus key factors sustaining the stable dominance of rootless submerged vegetation against free-floating plants. Consequently, N loading from e.g. agricultural runoff, groundwater or stormwater is assumed to trigger regime shifts to a dominance of free-floating plants and associated losses in ecosystem services.

## Introduction

Competition for nutrients or light strongly influence plant-plant interactions which, together with other factors such as allelopathy or susceptibility to herbivores or pathogens, can induce significant changes in community structure (e.g., Bornette and Puijalon, 2011; Le Bagousse-Pinguet, et al., 2012; Szabó et al., 2020). In aquatic systems, there is a trade-off among primary producers regarding light availability and nutrient uptake (Szabó et al., 2010). In larger open water bodies with high nutrient concentrations, phytoplankton usually dominates the primary production (Scheffer et al., 1993; Scheffer and Van Nes, 2007). However, in small lentic water bodies and in slow-flowing channels, free-floating plant dominance is frequently observed (Scheffer et al., 2003; Smith, 2014; Kazanjian et al., 2018) that suppresses submerged macrophytes by shading (Morris et al., 2003; Van Gerven et al., 2015). Phytoplankton and free-floating plants retrieve their nutrients from the open water and can thus be outcompeted at lower nutrient concentrations by submerged vegetation with access to sediment nutrients (Van Zuidam and Peeters, 2013; Szabó et al., 2022). Such submerged macrophyte dominance is associated with the highest number of ecosystem services compared to other vegetation types (Janssen et al., 2021).

Interestingly, reviewing the database of European surface waters revealed that rootless submerged vegetation also often dominates (Birk and Willby, 2010) despite its inferiority in the competition for light. Coontail (*Ceratophyllum demersum*) is one the most abundant, rootless submerged species in temperate water bodies (Hilt et al., 2018; Szabó et al., 2022) and often forms thick mats below the surface in small ponds and slow-flowing ditches (Lombardo, 2003). Field observations revealed a negative correlation between the occurrence of *C. demersum* and free-floating plants such as the duckweed species *Lemna gibba* (Feuchtmayr et al., 2009; Van Zuidam and Peeters, 2013; Koleszár et al., 2022). This raised the question how rootless species without access to sediment nutrients ever win against free-floating competitors that can shade out submerged macrophytes (Scheffer et al., 2003; Larson, 2007; Lu et al., 2013). Experiments along with a field survey showed that rootless submerged vegetation can sustain stable dominance against free-floating plants below a threshold nutrient concentration (Szabó et al., 2022). It was observed that beyond extremely high pH levels, concentrations of several nutrients [nitrogen (N), phosphorus (P), iron (Fe), manganese (Mn)] dropped below optimal levels for growth of free-floating plants (Szabó et al., 2022). However, according to Liebig’s law of the minimum, it is likely that free-floating plant growth is dictated by the scarcest resource. Accordingly, we tested the hypothesis that high pH and different nutrient deficiencies are not equally important for sustaining the stable dominance of rootless submerged macrophytes competing with free-floating plants. We performed co-culture and nutrient deficiency experiments to compare the single influence of N, P, Fe and Mn limitation as well as increased pH levels as potential mechanisms of growth retardation of free-floating *L. gibba* by the rootless submerged *C. demersum*. To rule out a potential allelopathic impact of *C. demersum* (Hilt and Gross, 2008), additional treatments were tested. In addition, monitoring data on the abundance of *C. demersum* in Hungarian water bodies and respective hydrochemical conditions were studied to complement laboratory results with field observations.

## Material and methods

Free-floating (*L. gibba*, subsequently termed *Lemna*) and rootless submerged plants (*C. demersum*, subsequently termed *Ceratophyllum*) plants were collected from canals near Nyíregyháza (NE Hungary, N 47.996376°, E 21.734152°). Plants were preincubated for two weeks under experimental conditions on a general purpose “BS” medium (Barko and Smart, 1985). This BS medium was supplemented by adding NH_4_NO_3_ to a final concentration of 5 mg N L^-1^. Phosphorus was added as K_2_HPO_4_ to a final concentration of 1.0 mg P L^-1^ and a supply of micronutrients was ensured by adding 0.1 mL L^-1^ TROPICA supplier micronutrient solution (final concentrations: 0.08 mg Fe L^-1^, 0.03 mg Mn L^-1^, 0.002 mg Zn L^-1^, 0.006 mg Cu L^-1^and 0.002 mg Mo L^-1^). The initial pH of the medium was adjusted to 7.3. Plant cultures were kept under 25°C, a photon flux density of 250 µmol m^-2^ s^-1^ and a 16 h light/8 h dark cycle.

### Co-culture experiment

**Figure 1:**
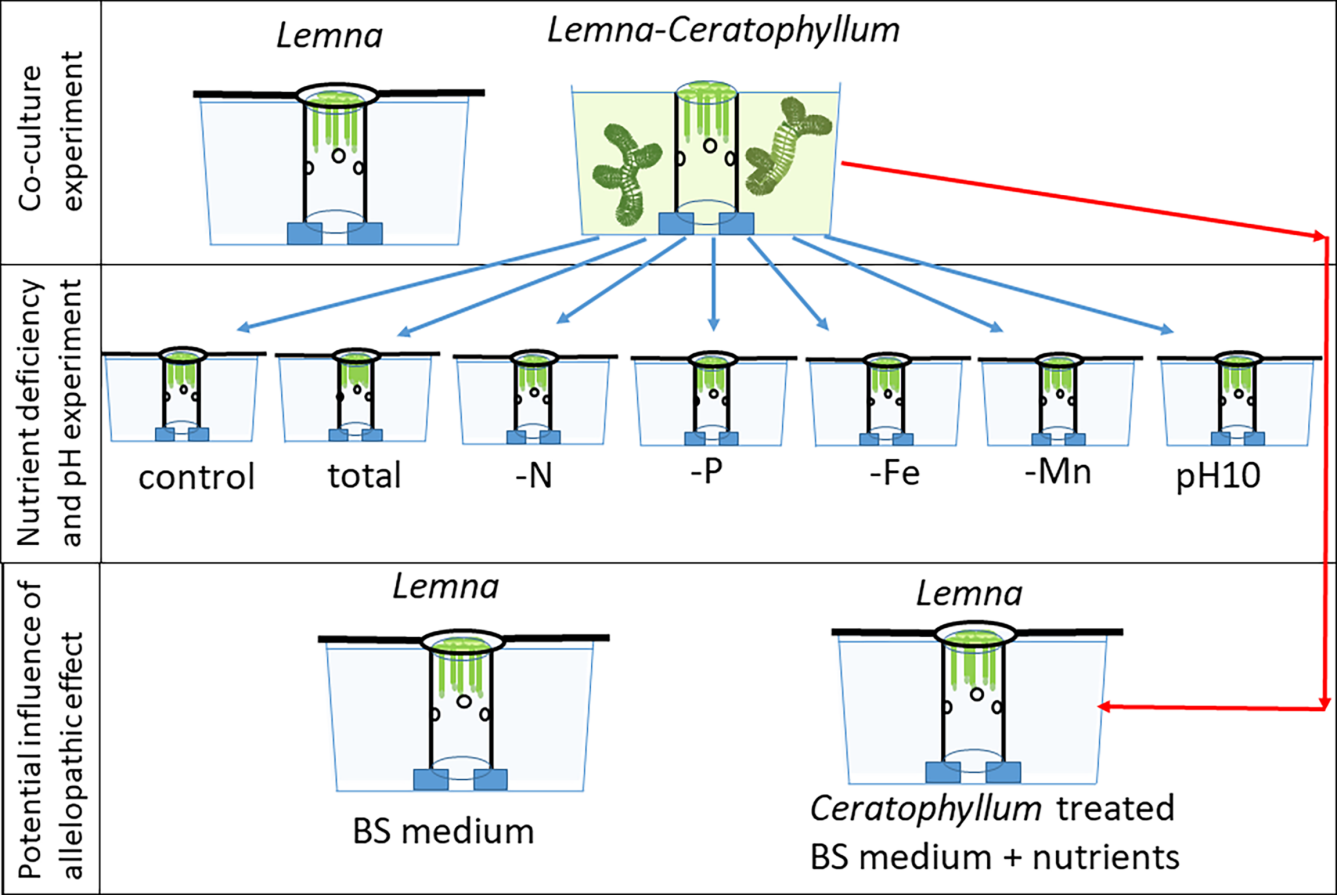
Setup of experiments testing the effect of the rootless submerged macrophyte *Ceratophyllum demersum* on the free-floating macrophyte *Lemna gibba* and disentangling the impact of different nutrient deficiencies, pH increases and allelopathic effects.

For the experiments, plastic tubes with a diameter of 5 cm were placed vertically in 2 L black plastic aquaria (**Figure 1**) serving as a duckweed enclosure (Szabó et al., 2003). For allowing nutrient transfer between the inner and outer medium, each tube had several holes. Portions of 100 ± 1 mg biomass (fresh weight) of *Lemna* (mean 83 ± 12 frond) were placed in the enclosures and co-cultured with 0 g (control) and 10 ± 0.1 g biomass of *C. demersum* plants (6-7 shoots, total length 122 - 135 cm) for 10 days. Co-cultures were grown in triplicate. In addition, 30 co-cultures were used for the subsequent nutrient deficiency experiment (**Figure 1**). *Ceratophyllum* shoots were grown outside of the *Lemna* enclosures. As soon as *Lemna* cover reached 100%, overcrowding was avoided by replacing the enclosure with a bigger one. In the control aquaria, the area outside the enclosures was completely covered by a grey plastic sheet to avoid algae growth. Biomass (fresh weight) of the *Lemna* fronds was measured on the 4th, 6th, 8th, and 10th days after blotting fronds on tissue to remove water. After 10 days, the *Lemna* plants were harvested, their dry weight (DW) was measured after drying to weight constancy at 80°C and relative growth rates (RGR) of the cultures were calculated: RGR = (lnDW_10_ – lnDW_0_)/t in which DW_12_ and DW_0_ are the dry weights after 10 days and at time zero, respectively, and t is the time in days. The RGR based on fresh weight (FW) was followed in time and was calculated for the 4th, 6th, 8th and 10th days of incubation: RGR10 = (lnFW_10_ – lnFW_8_)/2. Values of pH in the medium were recorded on every second day. Water samples were taken in triplicate at the beginning of the experiment and the 2^nd^, 4^th^, 6^th^ and 10^th^ day, filtered and analysed for concentrations of PO_4_^3-^, NO_3_^-^, NH_4_^+^, Fe, Mn, Zn, Cu and Mo. At the 10th day, the culture medium was gently decanted from 30 aquaria treated with *Ceratophyllum* (in total 50 L, put into one big container). After 24 hours of particle sedimentation at 5 °C in the dark, this water was decanted again and filtered through 10 µm pore diameter filters and analysed for its nutrient content as detailed above. This filtrate was used for the subsequent nutrient deficiency experiment as nutrient-depleted medium (**Table 1**, **Figure1**).

**Table 1:** Chemical composition of the culture medium in controls and in *Ceratophyllum demersum* treatments after 10 days (total nutrient depletion and high pH).

	Nutrient concentrations (mg L^-1^)				
Treatment	N	P	Fe	Mn	pH
Control	5.0	1.0	0.08	0.03	7.3
Total nutrient depletion and high pH	0.16	0.06	0.011	0.001	10.0
Only N deficiency	0.16	1.0	0.08	0.03	7.3
Only P deficiency	5.0	0.06	0.08	0.03	7.3
Only Fe deficiency	5.0	1.0	0.011	0.03	7.3
Only Mn deficiency	5.0	1.0	0.08	0.001	7.3
Ony high pH	5.0	1.0	0.08	0.03	10.0

For nutrient deficiency and high pH treatments, this medium was subsequently supplemented with nutrients and the pH adjusted so that it was only deficient in one nutrient or had only a high pH.

### Nutrient deficiency and high pH experiment

Two litre aquaria containing *Lemna* enclosures were covered by black foil on the sides to avoid light penetration and filled with 2 L of nutrient depleted medium from the previous co-culture experiment after adjusting the pH to 7.3 by adding 0.1 M HCl to the medium (apart for the high pH treatment, see below and **Table 1**). Nutrients were added to arrive at treatments with a depletion of only one respective nutrient (N, P, Fe, Mn) (**Table 1**; **Figure 1**). In control cultures, nutrient concentrations (N, P, Fe, Mn) and pH were adjusted as in the non-treated “BS” medium (**Table 1**). Nutrients were supplemented as NH_4_NO_3_, K_2_HPO_4_, FeSO_4_ and MnCl_2_. In *Lemna-Ceratophyllum* co-cultures, pH rose to 10.02. To measure the potential pH effect on the growth of *Lemna*, nutrient depleted media were supplemented with all nutrients (N, P, Fe, Mn) and the pH was adjusted daily to 10.02 by 0.2 M KOH. *Ceratophyllum* treated *L. gibba* fronds in nutrient-depleted state from the previous experiment (100 mg fresh weight each) were placed in the enclosures (5 cm in diameter) in each of the aquaria (four replicates per treatment). Similar to controls in the first experiment, the surface of the aquaria outside the enclosures was covered by grey plastic sheets to avoid algal growth. Overcrowding was avoided by replacing the enclosures with bigger ones (up to 5 times, up to 10 cm diameter). *Lemna* cultures were incubated for 14 days and biomass (fresh weight after blotting) of the fronds was measured on the 6^th^, 10^th^ and 14^th^ day. Relative growth rates (RGR_6-14_) were calculated for the period between the 6 and 14th day (Szabó et al., 2022). This period was chosen to avoid an influence of the initial lag phase in *Lemna* growth due to initial nutrient deficiency in the fronds from the previous experiment. After 14 days the *Lemna* plants were harvested. One part of the samples (50-100 mg) was used for chlorophyll determination, while the rest was used for determining dry weight and dry matter content (DMC) and subsequent analyses of C, N, P, Fe and Mn content (see below).

### Potential influence of allelopathic effects

For testing the possible allelopathic effects of *Ceratophyllum* on *Lemna* growth, 100 mg FW of preincubated *Lemna* fronds were grown in BS medium (5 mg N L^-1^, 1.0 mg P L^-1^, plus micronutrients) as control and in *Ceratophyllum* treated nutrient depleted media with nutrient concentrations (N, P, Fe, Mn) and pH adjusted as in the non-treated BS medium (**Table 1**). Both treatments (3 replicates each) were cultivated as described above. After 10 days the *Lemna* plants were harvested and their dry weight was determined to calculate RGR.

### Field surveys

Using the Database of Hungarian Surface Waters, we collected data from water bodies (246 locations) in which either *C. demersum* (n = 215) or *L. gibba* (n = 46) occurred between July and September in the period 2005 - 2019. We tested for correlations between the concentrations of dissolved inorganic nitrogen (DIN = NO_3_^-^ + NH_4_^+^) and ortho-phosphate in the water and the cover (in percent) of *Ceratophyllum*. To avoid an influence of other submerged plants, we selected only sites with a total cover of other submerged plants below the cover of *C. demersum* or a lower than 15% of the same procedure was applied for *L. gibba*. In addition, we took water samples from the upper 5 cm layer in *Ceratophyllum* dominated water bodies (N = 39) from drainage ditches, oxbow lakes and ponds in the North-eastern part of the Great Hungarian Plain. Samples were taken between 9 am and 3 pm in July and August and were analysed for pH and filtered through a filter with a 0.45 µm pore diameter. After adding 0.1 ml 67% (m/m) nitric acid into 20 mL of filtrate, iron and manganese content of the samples were measured by ICP OES.

### Analytical methods

Measurements of pH of the media were performed using a pH meter (Orion Start 2000). Nutrient (NH^+^_4_-N, NO_3_^–^N, PO_4_^3–^P) concentrations of filtered (0.45 µm) water samples were determined photometrically according to Hungarian standards (MSZ ISO 7150-1, 1992; MSZ EN ISO 6878, 2004; MSZ 1484-13, 2009). Concentrations of Fe, Mn Zn, Mo and Cu in the medium were determined by Agilent 8800 inductively coupled plasma mass spectrometry (ICP MS, Agilent Technologies, Waldbronn, Germany). Chlorophyll (chl) of *Lemna* fronds was extracted in 6 mL 95% ethanol for 24 h at 4°C in the dark. Concentration of chl a and b were measured by spectrophotometry (T80+ Spectrometer, PG Instruments Limited, UK) and calculated according to Lichtentaler (1987). Total carbon and nitrogen content in the *Lemna* plants were determined by dry combustion using a Vario Max Cube elemental analyzer (Elementar GMBH, Germany). Phosphorus, iron and manganese content of *Lemna* was measured by MPAS after acidic digestion using 5 mL of 67% (m/m) nitric acid and 5 mL 30% (m/m) hydrogen peroxide at 90°C for one hour. In all but controls and manganese depleted cultures, *Lemna* plant samples of the replicates were merged to arrive at sufficient biomass for analyses.

### Statistical methods

The impact of the different individual factors (nutrient deficiencies, high pH) on several dependent variables (biomass, RGR, total chl content, dry matter content) was tested by analysis of variance (ANOVA). Normal distribution of variables was checked by Kolmogorov-Smirnov tests. The homogeneity of the variances of the dependent variables was checked by Levene’s test. In Lemna-*Ceratophyllum* co-culture experiments, we used Pairwise Comparisons (PC) to test for significant differences between *Lemna* biomass and RGR_6-14_ in controls and *Ceratophyllum* treatments where mean differences (MD) ± SE were indicated. In nutrient deficiency experiments, we compared *Lemna* biomass (FW_14_), RGR_6-14_, and chl content among treatments using Tukey *post-hoc* tests. Field data were evaluated using a regression analysis with C. demersum density as independent and nutrient concentrations (DIN, PO_4_^3–^P) as dependent variable. All statistical analyses were performed using SPSS 16.0.

## Results

### Co-culture experiment

**Figure 2:**
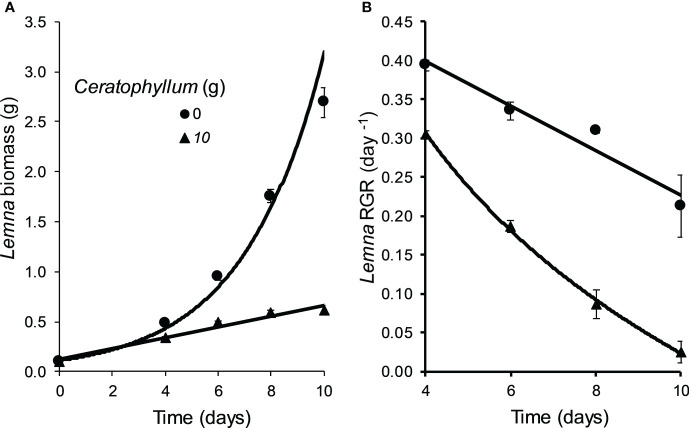
Effect of *Ceratophyllum demersum* on the biomass (fresh weight) **(A)** and the relative growth rate (RGR) **(B)** of *Lemna gibba* compared to treatments without *C. demersum* (means ± standard error, n = 3).

**Figure 3:**
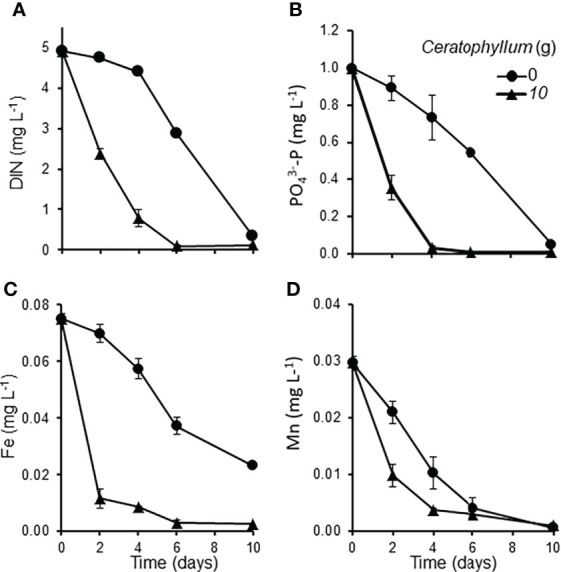
Nutrient concentrations in *Lemna-Ceratophyllum* co-cultures grown on static medium: **(A)** dissolved inorganic nitrogen (DIN: NH_4_^+^-N plus NO_3_^-^ -N), **(B)** PO_4_^3–^P, **(C)** total iron (Fe), **(D)** total manganese (Mn) (means ± standard error, n = 3).

The Control *Lemna* cultures co-cultivated without *Ceratophyllum* showed an exponential growth (RGR: 0.394 - 0.212 day^-1^). *Ceratophyllum* reduced the growth rate of *Lemna* significantly (MD 0.187 ± 0.042, P = 0.012). At the end of the experiment RGR of *Lemna* was reduced below 0.05 day^-1^ (**Figure 2**). The pH of treatments containing *Ceratophyllum* rose above 9.60 at the second day and reached 10.02 at the 10^th^ day. The concentration of dissolved inorganic nitrogen (NH^+^_4_-N and NO_3_^–^N) was decreased to 0.2 mg L^-1^ and concentrations of PO_4_^3–^P decreased below 0.02 mg L^-1^ within four days. Among the measured micronutrients (Fe, Mn, Zn, Cu, Mo) only the Fe and Mn concentrations showed a significant decline in treatments containing *Ceratophyllum* (**Figure 3**; **Table 1**).

### Nutrient deficiency and high pH experiment

**Figure 4:**
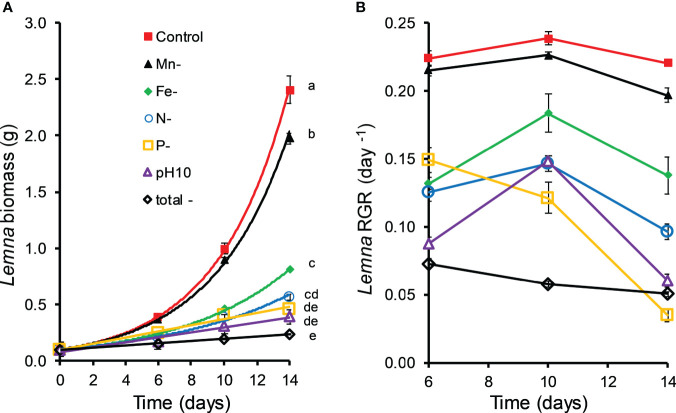
Biomass **(A)** and relative growth rates (RGR) **(B)** of *Lemna gibba* grown in nutrient deficient (N, P, Fe Mn) and high pH (10.0) media retrieved from co-culture experiments with *Ceratophyllum demersum* and supplemented with respective nutrients (see **Table 2**, means ± standard error, n = 4). Significant differences (oneway ANOVA, Tukey’s posthoc test, P < 0.05) among treatments are indicated with different lowercase letters.

Manganese deficiency had a slight but significant (MD 0.018 ± 0.009, P < 0.048) negative effect on the relative growth rate (RGR_6-14_) of *Lemna* (**Figures 4A, B**). The same holds for iron deficiency (MD 0.069 ± 0.009, P < 0.001). The impact of N deficiency was even stronger (MD 0.108 ± 0.009, P < 0.001) lowering influence. Treatments with P deficiency (MD 0.152 ± 0.009, P < 0.001) also showed a significantly reduced growth of *Lemna* which was similar to the growth in N deficient treatments. In treatments with all nutrients replaced, but a high pH of 10.02, the growth of *Lemna* was significantly (MD 0.126 ± 0.009, P < 0.001) lower in treatments with *C. demersum* than in controls. Total nutrient depletion together with a high pH (10.02) resulted in the strongest growth reduction of *Lemna* (**Figure 4**, MD 0.176 ± 0.009, P < 0.001). Iron, N, and P deficiency decreased the RGR_6-14_ of *Lemna* by 30%, 47% and 66%, respectively, and the high pH by 55% (ESM1).

**Figure 5:**
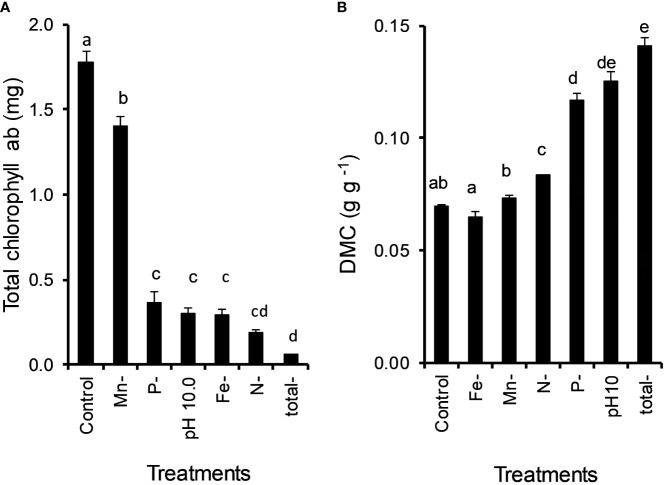
Effect of nutrient deficiencies (N, P, Fe, Mn) and high pH (10) caused by *Ceratophyllum demersum* on the total chlorophyll content **(A)** and dry matter content (DMC) **(B)** of *Lemna gibba* (means ± standard error, n = 4). Significant differences (oneway ANOVA, Tukey’s test, P < 0.05) among treatments are indicated with different lowercase letters.

All examined nutrient deficiencies (N, P, Fe, Mn) and high pH had significant effects on the chl content of *Lemna*. N deficiency of *Lemna* growth resulted in the strongest drop (MD 1.588 ± 0.066, P < 0.001) in chl content (**Figure 5A**). Mn, P, Fe and N deficiency decreased the chl content of *L. gibba* by 21%, 80%, 84% and 89%, respectively, and the alkaline pH by 83%.

Nutrient deficiency and high pH resulted 49-66% drop in elemental concentrations in *Lemna* (**Table 2**). The nitrogen concentration in N depleted *Lemna* fronds was reduced to a low level (1.3% DW). High pH resulted intermediate reduction in elemental concentrations of *Lemna* compared with nutrient deficiency (N, P, Fe, or Mn) or with total depleted fronds (**Table 2**).

**Table 2:** Elemental composition of *Lemna* fronds in nutrient deficiency and high pH experiment (data are in mg g^-1^ dry weight).

Treatment	N	P	N:P	Fe	Mn
Control	31.8 ± 1.94	1.89 ± 0.22	16.8	0.101 ± 0.008	0.048 ± 0.004
Only N depleted	12.6				
Only P depleted		0.97			
Only Fe depleted				0.035	
Only Mn depleted					0.017 ± 0.002
Only high pH	19.1	1.32	14.5	0.060	0.050
Total depletion	13.0	1.09	11.9	0.031	0.034

In control and in Mn depleted cultures, replicates were analysed separately (n = 4, means ± standard deviation), while in all other treatments, replicate *Lemna* samples were merged to arrive at sufficient biomass for analyses.

Nutrient deficiency and high pH significantly increased the dry matter content (DMC) of *Lemna* with the following order: N and P deficiency, high pH (by 20%, 68%, 81%). Total nutrient deficiency with high pH showed the highest DMC of *Lemna* (103%) (**Figure 5B**).

### Potential allelopathic effects

*Ceratophyllum* treated media with total nutrient supplement and adjusted pH had no significant (MD 0.013 ± 0.006, P = 0.087) effect on the RGR (calculated based on the dry weight) of *Lemna*.

### Field surveys

**Figure 6:**
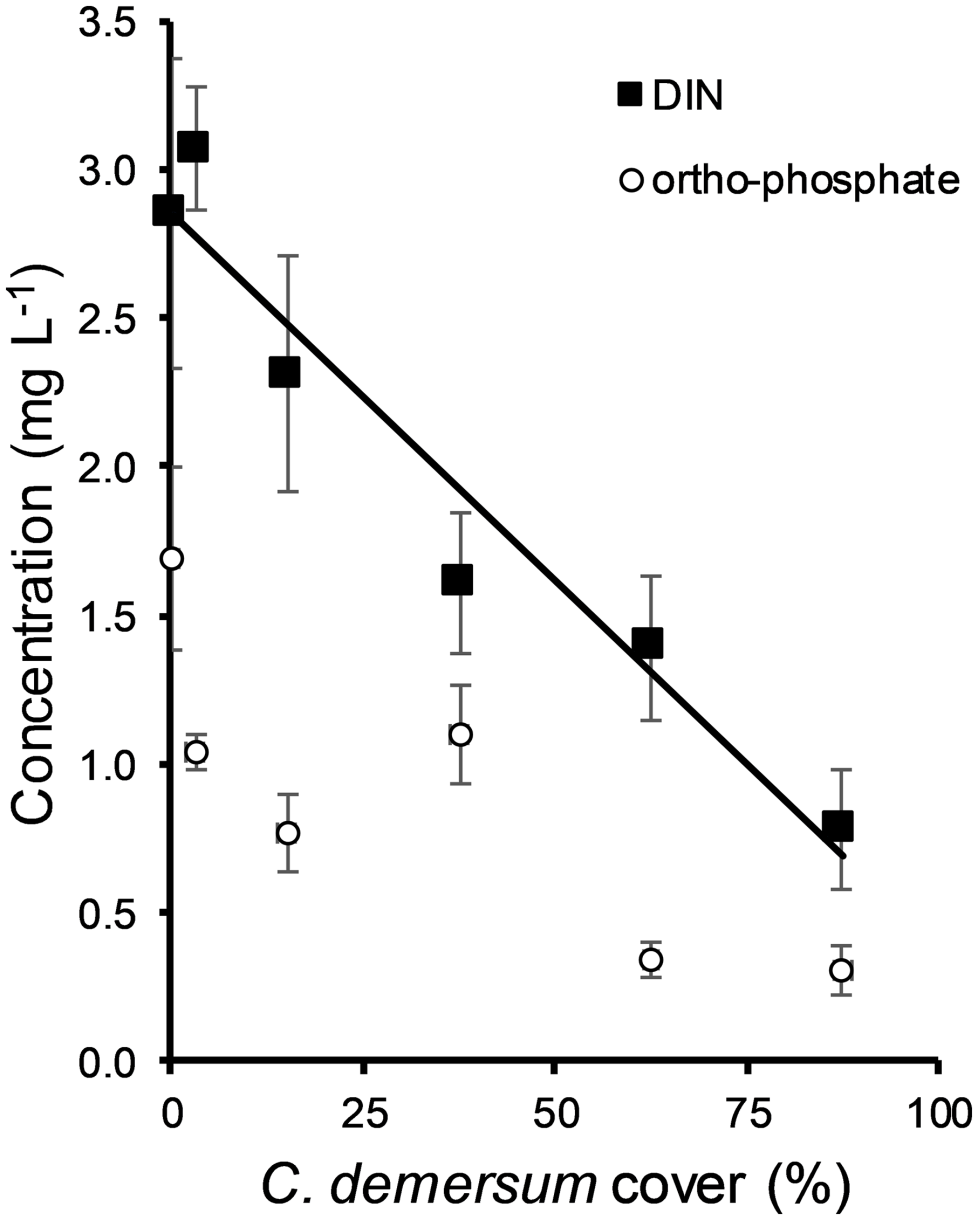
Correlation between the coverage of *Ceratophyllum demersum* and concentrations of dissolved inorganic nitrogen (DIN) and ortho-phosphate (means ± standard error) in Hungarian water bodies. Data were collected from the database of Hungarian surface waters (n = 173).

Based on the Database of Hungarian Surface Waters (n = 245) the concentrations of DIN and ortho-phosphate were 0.78 ± 0.22 mg L^-1^ (standard error) and 0.25 ± 0.09 mg L^-1^, respectively, in water bodies dominated by *Ceratophyllum* (coverage > 87.5%) (**Figure 6**). In 60% of the data, DIN concentrations were lower than 0.5 mg L ^-1^. DIN was negatively correlated with the cover of *Ceratophyllum* and this relationship was significant (ANOVA F = 8.141, P = 0.005). However, there was no significant relationship between the cover of *Ceratophyllum* and ortophosphate concentration (ANOVA, F = 0.285, P = 0.594).

In *Ceratophyllum* dominated (coverage > 87.5%) water bodies sampled in the northestern Great Hungarian Plain (N = 39), the pH of the water was high (10.18 ± 0.06), while Fe concentrations were moderately low (41.4 ± 6.7 µg L^-1^) and Mn concentrations were close to the detection limit (1.4 ± 0.2 µg L^-1^).

## Discussion

Our study disentangled the role of several potential factors that can limit the growth of free-floating plants when co-occurring with rootless submerged vegetation. While laboratory experiments pointed to a potentially strong influence of high pH and P, N or Fe deficiencies, field data revealed that P and Fe deficiencies were unlikely due to high concentrations in the water among dense stands of the rootless submerged plant *Ceratophyllum demersum*. Consequently, a high pH (10) and low N concentrations in water containing the rootless submerged *Ceratophyllum demersum* were indicated as key factors in their growth-inhibiting effect on *Lemna gibba*. Deficiencies in Mn played a lower role and allelopathy could be ruled out.

### Nutrient deficiency inhibiting the growth of free-floating plants

*Ceratophyllum* plants did reduce the concentrations of several nutrients (N, P, Fe, Mn) in the growth medium, but also alkalized the pH to high values (10.2 - 10.5). Low nutrient concentrations were probably not only caused by the uptake of macrophytes but also by their epiphytes (Koleszár et al., 2022). The reduced growth of floating plants in nutrient deficient media with high pH was relaxed after replacing nutrients and neutralizing the pH. Nutrient removal and high pH are thus explaining the inhibitory effect of rootless submerged plants on free-floating plants. *Ceratophyllum* and its epiphyton showed dissolved inorganic P- and N-uptake rates of 50 and 860 µg g^-1^ fresh weight day^-1^, respectively, during the first 4 days. These values were higher than those measured for rooted submerged macrophyte species such as *Myriophyllum spicatum* (Hilt and Lombardo, 2010). Even higher P uptake rates of up to 1 mg g^-1^ fresh weight day^-1^ have been measured for *C. demersum* during the first day, but declined exponentially with time (Lombardo and Cooke, 2003). Fast nutrient uptake by rootless submerged *Ceratophyllum* decreased inorganic N and P concentrations in the water below optimal levels for duckweed (Oscarson et al., 1989; Giblin et al., 2014; Petersen et al., 2021). Accordingly, the tissue N concentration in *Lemna* was reduced far below the range (22 - 38 mg N g^–1^) considered optimal for duckweed (Oscarson et al., 1989; Vermaat and Hanif, 1998). Low tissue N concentrations in the nutrient deficient *Lemna* cultures support the notion that N limitation was responsible for low growth rates (Oscarson et al., 1989; Demars and Edwards, 2007). In total nutrient depleted cultures, altough the tissue P concentration of *Lemna* was also quite low (13.0 mg P g^-1^), yet the plants were rather N than P limited, since their N:P ratio was still below the threshold indicating P limitation (25; Giblin et al., 2014). Iron concentrations in the Fe-deficient medium were lower than the minimal amount (0.028 mg L^-1^) reported for duckweed growth (Eyster, 1966 in Landolt and Kandeler, 1987). An allelopathic influence on *Lemna* was ruled out because *Ceratophyllum* treated media with total nutrient supplement did not significantly influence *Lemna* growth.

### High pH limiting the growth of *Lemna*

Under intensive underwater photosynthesis, hydrogen carbonate is taken up and *Ceratophyllum* plants alkalize the water by a release of OH^-^ ions (Pedersen et al., 2013). The resulting pH in our experiment was much higher than the range (pH 5 - 9) for optimal growth of *Lemna* species (McLay, 1976). This inhibitory mechanism may not only play a role in aquarium experiments (Szabó et al., 2005; Szabó et al., 2010; Szabó et al., 2022), but also under field conditions, because elevated pH values (>10) have frequently been found above dense stands of submerged vegetation (Frodge et al., 1990; Spencer et al., 1994; Stiers et al., 2011) and in *Ceratophyllum* stands in our field survey. Laboratory experiments suggest that alkaline pH (9.8 - 10.78) above dense stands of *Ceratophyllum* may be sufficient to completely inhibit the growth of floating plants. It is well known that alkaline pH does not only reduce the bioavailability of anions (ie. NO_3_^-^, PO_4_^3-^) (Loeppert et al., 1977; Ullrich-Eberius et al., 1981), but also leads to precipitation of phosphate (Otsuki and Wetzel, 1972), Fe and Mn (Stumm et al., 1960; Stumm and Morgan, 1995). In our experiments, high pH treatments resulted in reduced growth and lowered tissue N, P and Mn concentration and higher DMC in *Lemna*. Consequently, the higher pH treatment included multiple, potentially interactive factors and disentangling the impact of alkalinity alone was not possible.

### Differences among the impact of factors

Among potentially limiting factors, alkaline pH (84%) was the strongest, followed by P deficiency (81%), N deficiency (77%), and Fe deficiency (66%) based on biomass data of *Lemna*. Total chl content of *Lemna* was an even more sensitive indicator of limitation than growth rate implying that dense *Ceratophyllum* vegetation may eventually hinder *Lemna* growth completely. Each of these factors may intensify the negative effect of submerged plants on floating plants, increasing the chance for the occurrence of a hysteresis and thus alternative stable states (Scheffer et al., 2003; Scheffer and Van Nes, 2007).

Our experiments may overestimate the strength of the inhibitory effects, since many naturally occurring buffer mechanisms were excluded. Under field conditions, nutrient deficiency may be weakened by nutrient release from sediments, water movements or plant decomposition at the end of the growing season. Furthermore, top-down control of epiphytic algae by grazing snails may also affect the inhibitory effects (Yang et al., 2020; Koleszár et al., 2022). However, water chemistry data of the field survey revealed that above dense *Ceratophyllum* vegetation, P and Fe concentrations were sufficiently high, but dissolved inorganic N and Mn concentrations and pH were not in the range for optimal growth of *Lemna*. Experimental results nonetheless did not support that Mn could be a potential limiting factor. Consequently, low N levels and alkaline pH values generated by dense *Ceratophyllum* stands are suggested as key factors sustaining the stable dominance of rootless submerged vegetation against floating plants.

### Implications

A recent meta-analysis indicated that more than two thirds of aquatic freshwater ecosystems suffer from high nutrient loading (Nõges et al., 2016). Annual N flow to rivers is expected to increase rapidly from 64 Tg N yr^−1^ in 2000 to 84 Tg in 2050 (Bouwman et al., 2013), and N loading to freshwaters has increased relative to P (Peñuelas et al., 2013). Our finding that low N concentrations are a key factor for sustaining the stable dominance of rootless submerged vegetation against free-floating plants imply that higher N loading in the future increases the chance of regime shifts to a dominance of free-floating plants. This stable vegetation state is associated with significantly fewer ecosystem services (Janssen et al., 2021). Lowering N loading, which has been suggested to induce summer N limitation and control cyanobacterial blooms in polymictic lakes (Shatwell and Köhler, 2019), may thus also be an appropriate restoration measure for water bodies suffering from a dominance of free-floating macrophytes.

## Data availability statement

The raw data supporting the conclusions of this article will be made available by the authors, without undue reservation.

## Author contributions

SS, GK and GZ designed and performed the experiments. GK, GZ, PN and MB were responsible for chemical analytics. SS, GK and SH analyzed the data and wrote the manuscript. All authors contributed to the article and approved the submitted version.

## Funding

This study was financed by the Scientific Board of University of Nyíregyháza. GZ and GK received funding from the Doctoral School of Biological Sciences of the Hungarian University of Agriculture and Life Sciences.

## Conflict of interest

The authors declare that the research was conducted in the absence of any commercial or financial relationships that could be construed as a potential conflict of interest.

## Publisher’s note

All claims expressed in this article are solely those of the authors and do not necessarily represent those of their affiliated organizations, or those of the publisher, the editors and the reviewers. Any product that may be evaluated in this article, or claim that may be made by its manufacturer, is not guaranteed or endorsed by the publisher.
